# Application of hairless mouse strain to bioluminescence imaging of *Arc* expression in mouse brain

**DOI:** 10.1186/s12868-017-0335-6

**Published:** 2017-01-23

**Authors:** Hironori Izumi, Tetsuya Ishimoto, Hiroshi Yamamoto, Hisashi Mori

**Affiliations:** 1 Department of Molecular Neuroscience, Graduate School of Medicine and Pharmaceutical Sciences, University of Toyama, Toyama, 930-0194 Japan; 2 Division of Animal Resources and Development, Life Science Research Center, University of Toyama, Toyama, 930-0194 Japan

**Keywords:** Hairless, *Arc*, Development, Bioluminescence imaging

## Abstract

**Background:**

Bioluminescence imaging (BLI) is a powerful technique for monitoring the temporal and spatial dynamics of gene expression in the mouse brain. However, the black fur, skin pigmentation and hair regrowth after depilation of mouse interfere with BLI during developmental and daily examination. The aim of this study was to extend the application of *Arc*-*Luc* transgenic (Tg) mice to the BLI of neuronal activity in the mouse brain by introducing the hairless (HL) gene and to examine *Arc*-*Luc* expression at various developmental stages without interference from black fur, skin pigmentation, and hair regrowth.

**Results:**

The *Arc*-*Luc* Tg HL mice were established by crossing the Tg C57BL/6 mouse strain with the HL mouse strain. Under physiological and pathological conditions, BLI was performed to detect the signal intensity changes at various developmental stages and at an interval of <7 days. The established *Arc*-*Luc* Tg HL mice exhibited clear and stable photon signals from the brain without interference during development. After surgical monocular deprivation during visual-critical period, large signal intensity changes in bioluminescence were observed in the mouse visual cortex. Exposure of mice to a novel object changed the photon distribution in the caudal and rostral cerebral areas. The temporal pattern of kainic-acid-induced *Arc*-*Luc* expression showed biphasic changes in signal intensity over 24 h.

**Conclusions:**

This study showed the advantages of using the mutant HL gene in BLI of *Arc* expression in the mouse brain at various developmental stages. Thus, the use of the *Arc*-*Luc* Tg HL mice enabled the tracking of neuronal-activity-dependent processes over a wide range from a focal area to the entire brain area with various time windows.

**Electronic supplementary material:**

The online version of this article (doi:10.1186/s12868-017-0335-6) contains supplementary material, which is available to authorized users.

## Background

Bioluminescence imaging (BLI) is a powerful research technique based on detection of light emission produced by oxidation of luciferin by luciferase [1, 2]. The advantages of this technique in studies of small living animals are the following: high signal-to-noise (S/N) ratio with low background signal intensities, low limit and simplicity of detection, wide dynamic range of signal intensities, and applicability to genetic manipulation as well as continuous and quantitative analyses. BLI can also be applied repeatedly to the same animal, thus contributing to the reduction in the number of animals used. BLI can be applied to the detection of the biological signal intensity changes that affect the up- and down-regulations of transcription. Luciferase reporter animals are used in the research fields of inflammation [3], toxicology [4], oncology [5], angiogenesis [6], nutrition [7], and neurobiology [8]. Furthermore, split luciferase and bioluminescence resonance energy transfer (BRET) techniques have recently been developed for the detection of protein–protein interaction [9, 10].

Previously, we established a novel reporter transgenic (Tg) mouse strain, namely, *Arc*-*Luc* Tg mice, in which the firefly luciferase gene (*Luc*) is driven by the *Arc* promoter [11]. *Arc*, that is, the activity-regulated cytoskeleton-associated protein gene, is one of the immediate early genes (IEGs), which are highly responsive to various external stimuli in the central nervous system (CNS) [12, 13]. Using *Arc*-*Luc* Tg mice, we have successfully detected the bioluminescence signals of Arc-Luc in the adult mouse brain. The intensity of bioluminescence signals changed after physiological or pharmacological manipulations and the change well correlated with neuronal-activity-dependent *Arc* expression. Our *Arc*-*Luc* Tg mice were established using the C57BL/6 strain because this strain is one of the inbred strains most widely used in the fields of genetical engineering and neuroscience. However, its black fur, skin pigmentation, and hair regrowth after depilation interfere with BLI during development. Thus, the periods of BLI were restricted less than 24 h and at an interval of more than 4 weeks [11].

To extend the use of BLI at various mouse developmental stages, we have introduced a mutant HL gene into the original *Arc*-*Luc* Tg mice to overcome the above-mentioned problems. Here, we examined the availability of *Arc*-*Luc* Tg HL mice for BLI at various developmental stages under physiological and pathological conditions.

## Results

### In vivo imaging of Arc-Luc Tg HL mice

**Fig. 1:**
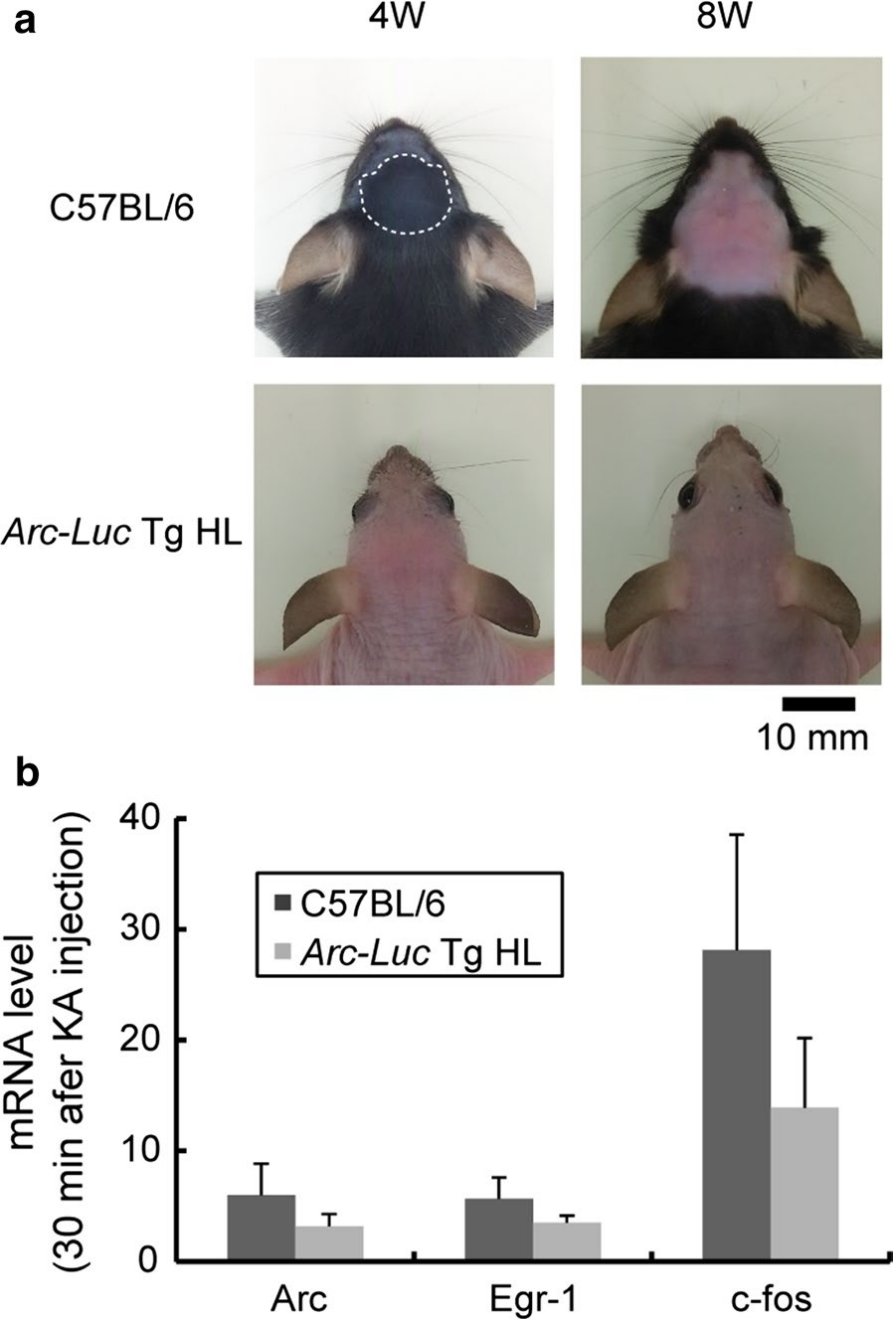
Comparison of pigmentation of head between C57BL/6 and *Arc*-*Luc* Tg HL mouse strains. **a** Dorsal views of head of C57BL/6 (*upper panels*) and *Arc*-*Luc* Tg HL (*lower panels*) mice at 4 (*left panels*) and 8 weeks of age (*right panels*). The photographs of the C57BL/6 mouse were taken after depilation. The C57BL/6 mouse at 8 weeks of age (*upper right panel*) and the *Arc*-*Luc* Tg HL mouse at both ages (*lower left* and *right panels*) are examples of suitable samples for BLI because of little pigmentation. The C57BL/6 mouse at 4 weeks of age (*upper left*) is an example of an unsuitable sample for BLI with skin pigmentation enclosed in a white dotted circle. *Scale bar* 10 mm. **b** Expression of IEGs in C57BL/6 and *Arc*-*Luc* Tg HL mice. Total RNA was extracted from the hippocampus 30 min after KA injection (n = 3, respectively). The changes in *Arc*, *Egr*-*1*, and *c*-*fos* expression levels were determined by quantitative RT-PCR analysis. The data represent mean ± SD

**Fig. 2:**
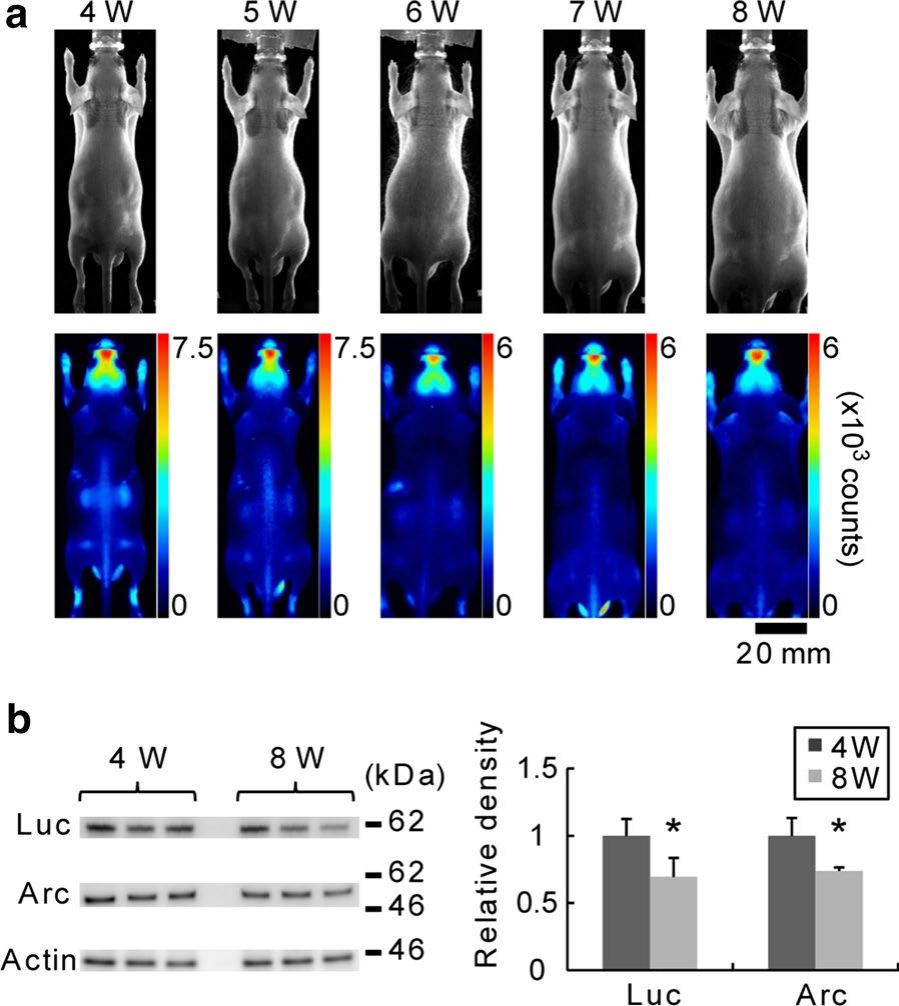
Noninvasive BLI using *Arc*-*Luc* Tg HL mouse. **a** Bright field (*upper panels*) and bioluminescence signal images (*lower panels*, pseudocolored, 0–7500 counts) of an individual *Arc*-*Luc* Tg HL mouse from 4 to 8 weeks of age. Bioluminescence signals in the *Arc*-*Luc* Tg HL mouse were obtained after luciferin injection (i.p., 200 mg/kg BW) under anaesthesia induced with isoflurane. *Scale bar* 20 mm. **b** Western blot analysis of proteins in the cerebral cortex was performed using anti-Luc (*upper panel*), anti-Arc (*middle panel*), and anti-actin (*lower panel*) antibodies (n = 3). Location of protein size markers are indicated on the right side. The optical density of each band was quantified using NIH ImageJ. The Luc and Arc protein expression levels normalized to actin protein expression level at 4 and 8 weeks of age are shown in the bar graph (*right panel*). The data represent mean ± SD. *p < 0.05; two-tailed Student’s *t* test

Previously, we successfully generated *Arc*-*Luc* Tg mice for the BLI of *Arc* expression [11]. However, the genetic background of the *Arc*-*Luc* Tg mice is C57BL/6, and the black fur and skin pigmentation of this strain attenuate photon emission at various developmental stages (Fig. 1a). Thus, we introduced the mutant HL gene into the original *Arc*-*Luc* Tg mice by crossing them with the HL mice. The established *Arc*-*Luc* Tg HL mice showed rapid induction of IEGs after kainic acid (KA) injection (*Arc*, *p* = 0.026; *Egr*-*1*, *p* = 0.023; *c*-*fos*, *p* = 0.049). The fold changes of these IEGs after KA injection in *Arc*-*Luc* Tg HL mice were the same as those in the C57BL/6 mouse strain (Fig. 1b, Additional file 1: Table S1, *Arc*, *p* = 0.16; *Egr*-*1*, *p* = 0.96; *c*-*fos*, *p* = 0.20). Using cooled CCD camera systems, we detected bioluminescence signals in areas around the nose, brain, paws, kidneys, and testes in the *Arc*-*Luc* Tg HL mice (Fig. 2a). The distribution of these signals was consistent with those previously reported for *Arc* expression and public database from transcriptome analysis [14]. The continuous BLI of an individual animal showed that the bioluminescence signal intensity in the brain gradually decreased from 4 to 8 weeks of age (Fig. 2a). Furthermore, we examined the expression levels of Luc and endogenous Arc proteins by western blot analysis and found that they were slightly higher in the cerebral cortex at 4 weeks of age than at 8 weeks of age (Fig. 2b, Additional file 1: Table S2). These developmental changes in protein expression levels correlated with the changes in bioluminescence signal intensity. These findings demonstrate that changes in the *Arc* expression level in the entire body during mouse development can be noninvasively detected by the visualization of *Arc*-*Luc* expression in *Arc*-*Luc* Tg HL mice.

### Monitoring responses to sensory stimuli

**Fig. 3:**
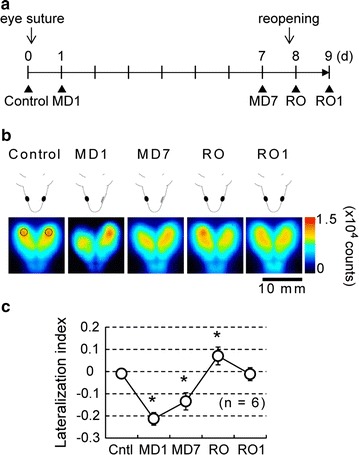
Laterality of bioluminescence signals from visual cortex after monocular deprivation (MD) by suturing and reopening surgery. **a** Experimental schedule for monocular deprivation and reopening and BLI. The time points of BLI are indicated with *black triangles*. **b** The obtained bioluminescence images (pseudocolored, 0–15,000 counts) before eye suture (Control on P21), 1 day after MD (MD1 on P22), 7 days after MD (MD7 on P28), immediately after reopening (RO on P29), and 1 day after RO (RO1). Mice were exposed to an intense light for 3 h before each imaging time point. The locations of regions of interest (ROIs) are enclosed in dotted circles in the control image, and the obtained bioluminescence signal intensities are used for the calculation of lateralization index (LI) shown in (**c**). *Scale bar* 10 mm. **c** Plots showing the LI under each condition shown in (**a**) (n = 6). LI was calculated using the formula (L − R)/(L + R), where L and R are the bioluminescence signal intensities of ROI in the regions contralateral (*left*) and ipsilateral (*right*) to the sutured eye, respectively. LI was nearly zero under the control condition (−0.009 ± 0.004), decreased below zero under MD conditions (MD1, −0.213 ± 0.012; MD7, −0.134 ± 0.015), and increased immediately at RO (RO, +0.070 ± 0.016), and finally returned close to the baseline at RO1 (RO1, −0.011 ± 0.016). The data represent mean ± SD. *p < 0.05; two-tailed Student’s *t* test

*Arc* is one of the IEGs, which are induced by various sensory stimuli. We previously reported the activity-dependent and plastic changes in Arc expression in the visual cortex of adult *Arc*-*Luc* Tg mice [11]. In this experiment, we extended the application of *Arc*-*Luc* Tg HL mice to continuous imaging at an interval of less than 7 days. We examined the pattern of changes in bioluminescence signal intensity during the development of mice in visual-critical period [Postnatal day 21 (P21)–P35] [15] with visual operation (Fig. 3a). The *Arc*-*Luc* Tg HL mice on P21 were monocularly deprived by eyelid suture. One day after monocular deprivation (MD1), the bioluminescence signal intensity significantly decreased in the brain region contralateral to the sutured eye after 3 h exposure to an intense light stimulus. Seven days after continuous monocular deprivation (MD7), the bioluminescence signal intensity in this region slightly increased (Fig. 3b). Furthermore, the reopening (RO) of the sutured eye followed by light exposure resulted in a marked increase in the bioluminescence signal intensity in this region (Fig. 3b). For the quantitative assessment of hemispheric dominance of Arc expression in the brain, we calculated lateralization index (LI), which is often used in functional magnetic resonance imaging (MRI) studies [16]. LI decreased to −0.21 (±0.03) on MD1, and it slightly increased on MD7 but remained below zero [−0.13 (±0.04)]. Moreover, LI further increased to +0.07 (±0.04) after RO and returned to about 0 one day after RO (Fig. 3c, Additional file 1: Table S3).

**Fig. 4:**
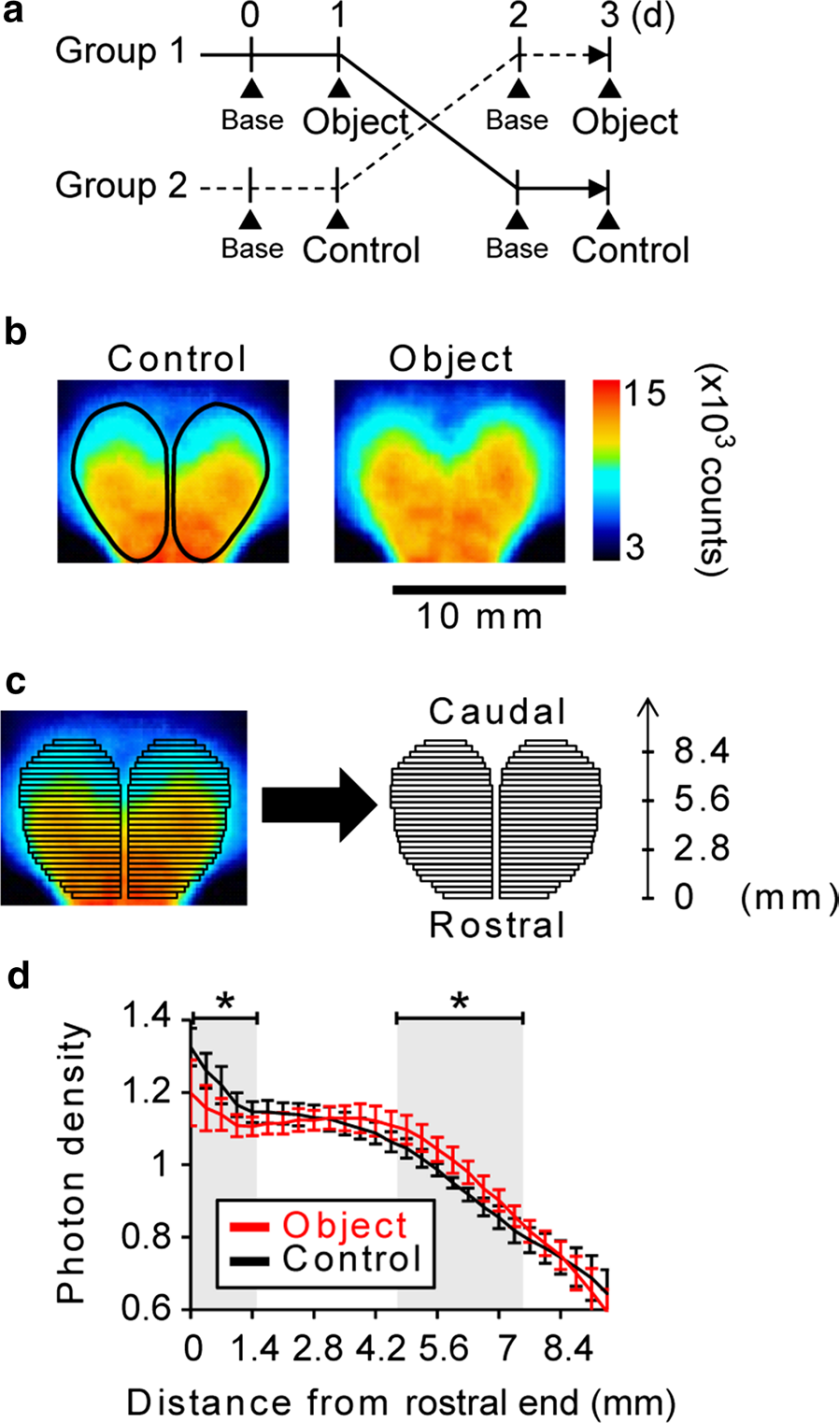
Bioluminescence signal intensity changes associated with OR task. **a** Experimental schedule for OR task and BLI. Bioluminescence signal intensity was measured in each mouse 6 h after putting a plastic cube into their home cage (Object). For the Control, no object was presented. BLI was also performed without any treatment 1 day before the OR task (Base). The time points of BLI are indicated with *black triangles*. **b** Representative images of bioluminescence signals (pseudocolored, 3000–15,000 counts) in the *Arc*-*Luc* Tg HL mouse brain at 4 weeks of age for the Object and Control. Location of ROIs in the forebrain is indicated in the Control image. *Scale bar* 10 mm. **c** Subdivision of ROI in forebrain into smaller rectangles at 0.35 mm intervals in rostral to caudal direction. **d** Distribution of bioluminescence signals associated with OR (n = 7). Profiles of photon density throughout the forebrain for object presentation (*red line*) and control condition (*black line*). Each value obtained from ROIs in (**b**) was normalized to the mean signal intensity (photon density = 1.0) in the whole forebrain. Significant differences between the control and OR conditions are shown in *gray* (0–1.4 mm and 4.55–7.35 mm from the rostral end) in the graph. The data represent mean ± SD. *p < 0.05; two-tailed Student’s *t* test

Spatial exploration in a novel environment induces Arc expression, and object and place recognition tasks induce the expression of some IEGs including Arc [17–19]. We carried out the evaluation of bioluminescence signal intensity changes associated with an object recognition (OR) task. Individually housed mice at 4 weeks of age were provided a novel plastic cube in their home cages (Fig. 4a). The object provided induced various exploratory behaviors of mice, such as approaching, sniffing, climbing, biting, and turning them over for several minutes. No statistically significant differences were detected in the total intensity of bioluminescence signals from the entire forebrain between the OR and control groups (Fig. 4b, *p* = 0.25). However, we observed marked differences in the distribution of bioluminescence signals from the brain between the two groups. We divided ROIs in the forebrain into 28 rectangular blocks in the rostral to caudal direction (Fig. 4c). The distribution of relative bioluminescence signal intensity normalized to the signal intensity corresponding to the entire-forebrain photon density showed a down-regulation of the signals from the rostral region (*p* < 0.05, at 0–1.4 mm from the rostral end) and the up-regulation of those from the posterior brain regions including the somatosensory, barrel, and visual cortices (*p* < 0.05, 4.55–7.35 mm from the rostral end) (Fig. 4d, Additional file 1: Tables S4 and S5). Therefore, we concluded that the detection of bioluminescence signals in the *Arc*-*Luc* Tg HL mice clearly provided detailed neuronal-activity-dependent *Arc* expression changes induced by sensory stimuli during development.

### Bioluminescence signal intensity changes after KA treatment

**Fig. 5:**
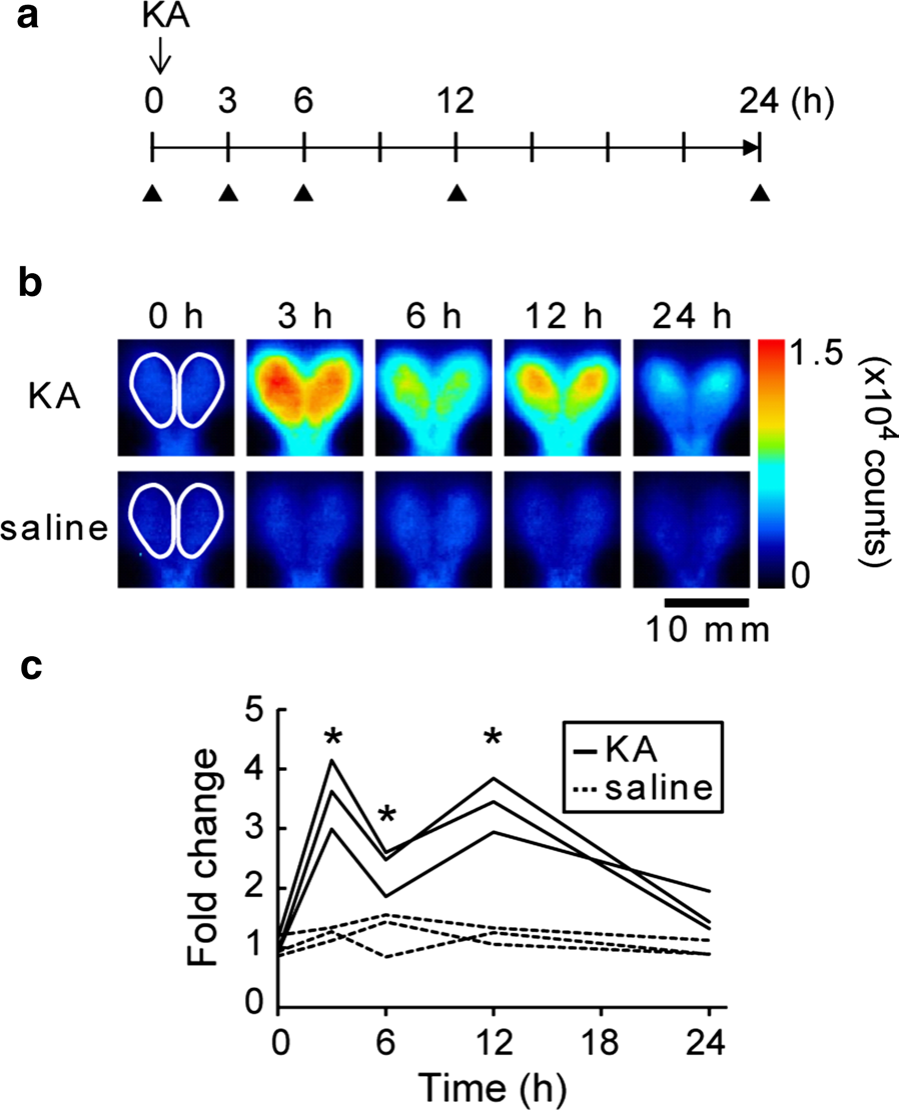
*Arc*-*Luc* expression level changes after KA injection at 4 weeks of age. **a** Experimental schedule for BLI after KA injection. The time points of BLI are indicated with *black triangles*. **b** Images (pseudocolored, 0–15,000 counts) from representative mice taken at 0 h (before injection) and 3, 6, 12 and 24 h after KA (*upper*) or saline (*lower*) injection. The locations of ROIs are indicated in the images at 0 h. *Scale bar*, 10 mm. **c** Changes in fold induction of bioluminescence signals within 24 h after KA injection (n = 3). *p < 0.05; two-tailed Student’s *t* test

KA is derived from a natural marine product from *Digenea simplex* [20] and is an excitatory amino acid receptor agonist selective to KA-type glutamate receptors. Acute treatment with KA is often carried out to produce a neurodegenerative disease model using laboratory animals and also induces *Arc* expression through synaptic activation. We reported the continuous increase in *Arc*-*Luc* expression level over 6 h after KA injection at the adult stage [11]. Here, we extend the examination of the temporal pattern of KA-induced *Arc* expression in the *Arc*-*Luc* Tg HL mice at 4 weeks of age (Fig. 5a). BLI was conducted after intraperitoneal KA injection (20 mg/kg BW) (Fig. 5b, c, Additional file 1: Table S6). The bioluminescence signal intensity increased three to fourfold within 3 h after KA injection, and then decreased to about twofold at 6 h. Furthermore, a second increase was detected in the posterior region at 12 h and the intensity finally returned close to the baseline at 24 h; this signal distribution pattern was different from that in the control saline-treated group. These results obtained from the *Arc*-*Luc* Tg HL mice suggest the advantages of temporal and quantitative evaluations of the effect of pharmacological manipulation in vivo at various developmental stages without interferences from fur and skin pigmentation.

## Discussion

BLI is a noninvasive, simple, and quantitative method to evaluate brain function. However, the BLI of the mouse brain using the C57BL/6 strain at various developmental stages has some problems such as light attenuation due to its black fur and skin pigmentation and the limited imaging period and interval caused by the hair regrowth after depilation. To overcome these problems, we established the *Arc*-*Luc* Tg HL mouse strain for imaging neuronal-activity-dependent *Arc* expression at various developmental stages. We examined the reliability of this new mouse strain for investigating neuronal-activity-dependent *Arc* expression processes.

The *Arc*-*Luc* Tg HL mice have several advantages that make them useful for investigating the neuronal-activity-dependent changes during development. First, we can acquire stable bioluminescence signals from the brain without interferences after 3 weeks of age. Albino mice are also useful for BLI without shaving, but there remains the problem of light absorbance by white fur [21]. The application of our novel mouse strain to BLI is valuable in terms of the increase in the intensity of bioluminescence signals per specified time interval. As shown in Fig. 2, comparison of bioluminescence signal intensities at various developmental stages of the same animal enabled the detection of age-related changes in Arc expression level. Second, we can track bioluminescence signals daily and weekly. Because skin pigmentation occurs 5 days after depilation of the C57BL/6 mouse strain, we cannot measure bioluminescence signals in the original *Arc*-*Luc* Tg mice for more than five consecutive days. We have to wait at least 4 weeks for the full regrowth of hair for the next BLI. The *Arc*-*Luc* Tg HL mice on the other hand can be used daily and weekly to detect time-dependent changes in Arc expression level in the brain by BLI (Figs. 2, 3, 4). Third, because the use of *Arc*-*Luc* Tg HL mice enables BLI for extended periods using the same animal, we can investigate the life-long effects of damage incurred from the early stages to the adulthood stage by BLI of *Arc* expression using fewer mice. However, despite these advantages of applying the *Arc*-*Luc* Tg HL mice to BLI, imaging at 1 or 2 weeks of age remains unfeasible because the hair loss in *Arc*-*Luc* Tg HL mice starts approximately on P14. There are several reports about the genes related to the regulation of hair growth and hair cycle [22–24], but the molecular mechanism underlying the initiation of first hair growth is as yet not fully understood. Further improvement of BLI to overcome this problem is necessary.

The Arc expression level in the brain slightly decreased from the early to adult stages (Fig. 2). Furthermore, we observed that the bioluminescence signal intensity of Arc-Luc in the submandibular gland increased with growth (data not shown). These findings suggest that Arc expression in mice was driven in age-dependent and tissue-specific manners.

In this study, BLI along with surgical monocular deprivation showed rapid changes in the bioluminescence signal intensity in the region contralateral to the deprived eye on MD1 and the subsequent recovery on MD7 (Fig. 3). Furthermore, continuous BLI showed the transient up-regulation of signals in the region contralateral to the deprived eye by reopening (LI shifted to over zero on the day of RO). These changes in bioluminescence signal intensity can be explained by reduced neuronal activity in contralateral visual cortex (MD1), recovery of neuronal activity in contralateral visual cortex by increase of the input from open eye (MD7), and increased neuronal activity in contralateral visual cortex by reopening of deprived eye (RO). Some reports indicate the changes in Arc expression during visual-critical period in the visual cortex [25, 26]. Taken together with these previous reports, our results suggest that bioluminescence signal intensity changes caused by visual operation are induced by up- and down regulation of neuronal activity in visual cortex at a critical period.

Arc is induced by the OR task and spatial exploration in novel environments [17–19]. On the basis of this finding, we examined the signal intensity changes of Arc associated with the OR task. We successfully detected an increase in bioluminescence signal intensity in the posterior region of the brain including the somatosensory, barrel, and visual cortices of the mice after exposure to the novel object in their home cages (Fig. 4). Our findings suggest that a novel object is mainly processed using the senses of touch and sight. Although it is generally believed that mice mainly use the senses of smell and touch for OR, visual information also must be used for OR. Furthermore, the use of *Arc*-*Luc* Tg HL mice in combination with behavioral analysis may lead to the development of a technique for monitoring the neuronal activity in higher brain functions, such as recognition, emotion, and learning and memory.

In Fig. 5, we detected biphasic bioluminescence signal changes with a 9 h interval between the early (3 h) and late phases (12 h) after KA injection in the *Arc*-*Luc* Tg HL mice. The rapid and protein-synthesis-independent transcription of *Arc* as an IEG is based on ‘promoter proximal Pol II stalling’ [27] and a specific enhancer with highly responsive to neuronal activity is located in the *Arc* promoter [28]. On the other hand, *Arc* expression under the regulation of new protein synthesis resulted in a late response [29, 30]. Actually, several studies showed a biphasic Arc induction with an 8–10 h interval and the necessity of protein synthesis for late induction of Arc after an electroconvulsive shock [31] and contextual fear conditioning [32]. Considering the regulation of *Arc* expression, the same molecular mechanism may explain our results. However, an increase in bioluminescence signal intensity after KA injection might be a result of greater entry of d-luciferin caused by the disruption of blood brain barrier. Further study is required for the evaluation of d-luciferin penetrance after status epilepticus.

## Conclusions

We have shown the advantages of an application of the HL gene to BLI of the mouse brain in this study. Without interferences, the *Arc*-*Luc* Tg HL mice exhibited clear and stable photon signals and enabled the tracking of *Arc* expression under physiological and pathological conditions during development and at a short time interval. These results indicate that this mouse strain can be used for rapid and quantitative assessments of neuronal-activity-dependent processes in the mouse brain over a wide range from a focal area to the entire brain area with various time windows.

## Methods

### Animals

The experimental procedures used in this study were carried out in accordance with the Guidelines for the Care and Use of Laboratory Animals of the University of Toyama, and were approved by the Animal Experiment Committee of University of Toyama (Authorization No. A2011med-13). This study complies with the ARRIVE (Animal Research: Reporting In Vivo Experiments) guidelines. *Arc*-*Luc* Tg HL mice were established by crossing *Arc*-*Luc* Tg C57BL/6 mice [11] with a hairless mouse strain (Hos:HRM, Hoshino Laboratory Animals, Inc., Japan). All the animals were maintained under standard laboratory conditions (12–12 h/light–dark cycle with light on at 7:00 am; room temperature, 22 ± 2 °C) in the Laboratory Animal Resource Center of the University of Toyama. The *Arc*-*Luc* Tg HL mice were genotyped by PCR for the firefly luciferase gene (*Luc2*, Promega, Madison, WI, USA) and Southern blot analysis for *Arc*-*Luc* as previously described [11]. The heterozygous *Arc*-*Luc* Tg HL mice were bred and the resulting heterozygous and homozygous *Arc*-*Luc* Tg HL mice were used in this study.

### Quantitative RT-PCR analysis

Total RNA was isolated from adult C57BL/6 and *Arc*-*Luc* Tg HL mouse hippocampi 30 min after KA injection [20 mg/kg BW, intraperitoneally (i.p.)] with TRIzol Reagent (Invitrogen, Carlsbad, CA, USA). Saline was injected as a control condition. Total RNA (1 μg) was reverse-transcribed using ReverTra Ace (Toyobo, Tokyo, Japan) in accordance with the manufacturer’s protocols. Quantitative PCR amplification was performed using the Mx3005P qPCR system (Agilent Technologies, Santa Clara, CA, USA) with Thunderbird SYBR qPCR Mix (Toyobo, Tokyo, Japan) for comparative quantification. The Ct of glyceraldehyde-3-phosphate dehydrogenase (GAPDH) was used for the calculation of the relative quantities of target genes. The following primers were used: 5′-GAGAGCTGAAAGGGTTGCAC-3′ and 5′-GCCTTGATGGACTTCTTCCA-3′ for *Arc*, 5′-TATACTGGCCGCTTCTCCCT-3′ and 5′-AGAGGTCGGAGGATTGGTCA-3′ for *Egr*-*1*, 5′-CTGAGATTGCCAATCTGCTG-3′ and 5′-AGACATCTCCTCTGGGAAGC-3′ for *c*-*fos* and 5′-ACAGTCCATGCCATCACTGC-3′ and 5′-TAGGAACACGGAAGGCCATG-3′ for *GAPDH*.

### BLI of live mice

In vivo BLI was performed as described previously [11]. Briefly, individually housed mice were anaesthetized by inhalation of isoflurane (1.5% in air) and intraperitoneally (i.p.) injected with d-luciferin (AAT Bioquest, Sunnyvale, CA, USA) dissolved in phosphate-buffered saline (PBS, pH 7.4) at 200 mg/kg body weight (BW). Ten minutes after luciferin injection, bioluminescence signal intensity was repeatedly measured for 30–120 s using an in vivo imaging system (Clairvivo OPT; Shimadzu Co., Kyoto, Japan) with 4 × 4 binning and without using an optical filter or excitation LED lights, as reported previously [11]. Bioluminescence signal intensity from captured images was calculated by region of interest (ROI) analysis using NIH ImageJ. Background bioluminescence signal intensity in non-Tg mice was subtracted from all values. Data were expressed as the mean number of photons per pixel (counts) in the ROI.

### Western blot analysis

Deeply anaesthetized mice (sodium pentobarbital, 150 mg/kg BW, i.p. injected) were transcardially perfused with ice-cold PBS. The cerebral cortex was dissected and homogenized in ice-cold Mammalian Tissue Extraction Reagent (Thermo Scientific, Rochford, IL, USA) supplemented with 1 mM phenylmethylsulfonyl fluoride and a protease inhibitor cocktail (Roche Diagnostics, Mannheim, Germany). The extracted proteins (10 μg) separated by SDS-PAGE were transferred to polyvinylidene difluoride (PVDF) membranes (GE Healthcare, Little Chalfont, Buckinghamshire, UK). After blocking with 5% skim milk in PBS containing 0.1% Tween20, the membranes were incubated with the appropriate primary antibodies overnight at 4 °C. The membranes were washed and incubated with the appropriate horseradish peroxidase (HRP)-conjugated secondary antibodies. Protein bands were visualized using the ECL Plus Western Blotting Detection Reagents (GE Healthcare, Little Chalfont, Buckinghamshire, UK) and measured using the ImageQuant LAS-4000 system (GE Healthcare, Little Chalfont, Buckinghamshire, UK).

The following antibodies were used: rabbit anti-Arc (1:1000, Synaptic Systems, Goettingen, Germany), goat anti-luciferase (1:1000, Promega, Fitchburg, WI, USA), rabbit anti-actin (1:2000 Santa Cruz Biotechnology, Dallas, TX, USA), HRP-conjugated goat anti-rabbit (1:2000, Bio-Rad, Hercules, CA, USA), and HRP-conjugated rabbit anti-goat (1:2000, Invitrogen, Camarillo, CA, USA) antibodies.

### Surgical monocular deprivation

Mice were anaesthetized with sodium pentobarbital (50 mg/kg BW i.p. injected) and the eyelids were sutured using 6-0 vicryl after BLI on postnatal day 21 (P21). Monocular deprivation (MD) was continued for 7 days and checked for complete eye closure, and the sutured eye was reopened on P29. Animals with signs of injury or infection were excluded from this experiment. BLI was conducted after exposure to an intense light for 3 h at each time point (Fig. 3). LI [16] was calculated using the equation LI = (L − R)/(L + R), where L and R are the signal intensities of the ROIs in the brain regions contralateral (left) and ipsilateral (right) to the deprived eye, respectively. A positive LI indicates deprived eye dominance, whereas a negative LI indicates nondeprived eye dominance.

### Exposure to novel object

Individually caged mice at 4 weeks of age were used as subjects. The mice were divided into the object recognition (OR) and control groups. On the first day, bioluminescence signal intensity was measured in each mouse without any treatments. The next day, the mice in the OR group were exposed to a novel cube in their home cages for 6 h followed by BLI. The cube was a plastic Lego block. Mice in the control group were kept in their home cages until signal intensity measurement. Then, the mice in the OR and control groups were switched and treated as described above. Small rectangular ROIs (2.5–5.3 mm width × 0.35 mm length) in the rostral to caudal direction were set on a template of the forebrain observed in the acquired photon image (Fig. 4b, c). The photon density in each rectangular ROI was calculated and normalized to the mean photon density in the entire forebrain.

### Exposure to KA

Mice at 4 weeks of age were separated into individual cages and i.p. injected with KA (20 mg/kg BW) dissolved in saline. KA-treated mice showed limbic motor seizure for 1–3 h. Control mice were injected with the same volume of saline. We measured bioluminescence signal intensity before (at 0 h) and after KA injection (at 3, 6, 12 and 24 h). The area enclosed in a white line corresponding to the entire brain (Fig. 5b) was used as a ROI. The bioluminescence signal changes in each group were expressed as a fold induction based on the signal intensity obtained at 0 h.

### Statistical analysis

All values are presented as mean ± standard deviation (SD). The statistical significance of differences was determined by two-tailed Student’s *t* test. Values of *p* < 0.05 were considered significant.

## Additional file

**Additional file 1: Table S1.** IEGs mRNA levels relative to GAPDH at 30 min after KA injection in Fig. 1b. **Table S2,** Relative density of Luc and Arc proteins in Fig. 2b. **Table S3,** LI values under visual operation in Fig. 3c. **Table S4,** Photon density throughout the forebrain of Control in Fig. 4d. **Table S5,** Photon density throughout the forebrain of OR in Fig. 4d. **Table S6,** Fold induction of bioluminescence signals by KA in Fig. 5c.

## Abbreviations

Arc: activity-regulated cytoskeleton-associated protein gene
BLI: bioluminescence imaging
BRET: bioluminescence resonance energy transfer
BW: body weight
CNS: central nervous system
HL: hairless
IEGs: immediate early genes
i.p.: intraperitoneally
KA: kainic acid
LI: lateralization index
Luc: luciferase
MD: monocular deprivation
MRI: magnetic resonance imaging
OR: object recognition
PBS: phosphate-buffered saline
PVDF: polyvinylidene difluoride
RO: reopening
ROI: region of interest
SD: standard deviation
S/N: signal-to-noise
Tg: transgenic
