# Supplementary material for: Application of hairless mouse strain to bioluminescence imaging of Arc expression in mouse brain
Source: BMC Neurosci. 2017 Jan 23;18:18. doi: 10.1186/s12868-017-0335-6 (PMC5260114; doi:10.1186/s12868-017-0335-6)
Supplement: Supplementary file 1 — Additional file 1: Table S1. IEGs mRNA levels relative to GAPDH at 30 min after KA injection in Fig. 1b. Table S2, Relative density of Luc and Arc proteins in Fig. 2b. Table S3, LI values under visual operation in Fig. 3c. Table S4, Photon density throughout the forebrain of Control in Fig. 4d. Table S5, Photon density throughout the forebrain of OR in Fig. 4d. Table S6, Fold induction of bioluminescence signals by KA in Fig. 5c. [file 12868_2017_335_MOESM1_ESM.docx]

**Data and materials**

Table S1. IEGs mRNA levels relative to GAPDH at 30 min after KA injection in Fig. 1b

| **Sample** | **Arc** | **Egr-1** | **c-fos** |
| --- | --- | --- | --- |
| B6-1 | 6.21187 | 3.05957 | 34.06621 |
| B6-2 | 3.51546 | 2.61720 | 14.53122 |
| B6-3 | 8.82393 | 5.66561 | 43.37697 |
| *Arc-Luc* Tg HL-1 | 4.45435 | 4.66075 | 23.63965 |
| *Arc-Luc* Tg HL-2 | 2.22696 | 2.26266 | 6.20512 |
| *Arc-Luc* Tg HL-3 | 3.29008 | 4.24927 | 15.47345 |

Table S2. Relative density of Luc and Arc proteins in Fig. 2b

| Sample | Relative density (Luc) | Relative density (Arc) |
| --- | --- | --- |
| 4w-1 | 1.28139 | 0.86018 |
| 4w-2 | 1.00286 | 0.70949 |
| 4w-3 | 1.14430 | 0.74263 |
| 8w-1 | 0.92423 | 0.56030 |
| 8w-2 | 0.76563 | 0.54360 |
| 8w-3 | 0.61118 | 0.52101 |

Table S3. LI values under visual operation in Fig. 3c

| Sample No. | Cntrl | MD1 | MD7 | RO | RO1 |
| --- | --- | --- | --- | --- | --- |
| 1 | 0.00039 | -0.00608 | 0.01337 | 0.02160 | -0.01404 |
| 2 | -0.01909 | 0.01775 | -0.04268 | -0.01459 | -0.00043 |
| 3 | -0.05632 | 0.00252 | -0.01650 | -0.03118 | -0.01990 |
| 4 | -0.03521 | -0.00920 | 0.01387 | -0.00094 | - |
| 5 | -0.01865 | 0.02181 | -0.00786 | -0.00999 | - |
| 6 | -0.00005 | 0.01486 | -0.01162 | -0.01525 | - |

Table S4. Photon density throughout the forebrain of Control in Fig. 4d

| Distance | mouse 1 | mouse 2 | mouse 3 | mouse 4 | mouse 5 | mouse 6 | mouse 7 |
| --- | --- | --- | --- | --- | --- | --- | --- |
| 0 | 1.31888 | 1.36675 | 1.35424 | 1.34162 | 1.35017 | 1.33090 | 1.21265 |
| 0.35 | 1.26795 | 1.30535 | 1.27236 | 1.27576 | 1.27120 | 1.26997 | 1.15385 |
| 0.7 | 1.23543 | 1.27428 | 1.21788 | 1.23664 | 1.22094 | 1.24447 | 1.11067 |
| 1.05 | 1.17344 | 1.19590 | 1.17014 | 1.16782 | 1.15548 | 1.19961 | 1.10140 |
| 1.4 | 1.15694 | 1.15601 | 1.15352 | 1.15418 | 1.11565 | 1.18461 | 1.09887 |
| 1.75 | 1.15804 | 1.14711 | 1.15686 | 1.15946 | 1.09389 | 1.19083 | 1.11791 |
| 2.1 | 1.15366 | 1.14204 | 1.14674 | 1.16221 | 1.08701 | 1.17898 | 1.12198 |
| 2.45 | 1.15126 | 1.13338 | 1.13714 | 1.16508 | 1.08080 | 1.17703 | 1.12561 |
| 2.8 | 1.14649 | 1.12847 | 1.12546 | 1.16128 | 1.06189 | 1.16612 | 1.12278 |
| 3.15 | 1.14190 | 1.12712 | 1.10843 | 1.15909 | 1.06844 | 1.16506 | 1.11632 |
| 3.5 | 1.12295 | 1.11221 | 1.09584 | 1.13975 | 1.05708 | 1.15350 | 1.11402 |
| 3.85 | 1.11409 | 1.09052 | 1.07936 | 1.11656 | 1.05137 | 1.13616 | 1.11237 |
| 4.2 | 1.11154 | 1.06853 | 1.07141 | 1.10183 | 1.03492 | 1.11772 | 1.10612 |
| 4.55 | 1.08882 | 1.03684 | 1.05112 | 1.08123 | 1.01865 | 1.08607 | 1.08184 |
| 4.9 | 1.07589 | 1.02132 | 1.02695 | 1.05919 | 1.00601 | 1.06702 | 1.06206 |
| 5.25 | 1.04702 | 0.99844 | 0.99834 | 1.02884 | 0.98249 | 1.03315 | 1.03797 |
| 5.6 | 0.99803 | 0.97164 | 0.96589 | 0.98795 | 0.97195 | 1.00151 | 1.01153 |
| 5.95 | 0.95836 | 0.92946 | 0.93117 | 0.95304 | 0.95917 | 0.95337 | 0.96879 |
| 6.3 | 0.92596 | 0.90155 | 0.89536 | 0.91277 | 0.93734 | 0.91258 | 0.94326 |
| 6.65 | 0.88036 | 0.87063 | 0.86412 | 0.87425 | 0.92462 | 0.86346 | 0.91129 |
| 7 | 0.83975 | 0.84261 | 0.83356 | 0.83554 | 0.91222 | 0.83535 | 0.88799 |
| 7.35 | 0.79497 | 0.80291 | 0.82123 | 0.79674 | 0.88279 | 0.78957 | 0.84316 |
| 7.7 | 0.77036 | 0.78025 | 0.80953 | 0.76029 | 0.84503 | 0.75585 | 0.82565 |
| 8.05 | 0.73647 | 0.76178 | 0.79828 | 0.73690 | 0.82430 | 0.72140 | 0.81199 |
| 8.4 | 0.71202 | 0.74269 | 0.77294 | 0.70674 | 0.81015 | 0.68518 | 0.78399 |
| 8.75 | 0.67478 | 0.72700 | 0.74556 | 0.67894 | 0.78215 | 0.64461 | 0.77300 |
| 9.1 | 0.65116 | 0.69483 | 0.71613 | 0.63970 | 0.76373 | 0.59476 | 0.75272 |
| 9.45 | 0.59348 | 0.67038 | 0.68044 | 0.60660 | 0.73058 | 0.54117 | 0.69019 |

Table S5. Photon density throughout the forebrain of OR in Fig. 4d

| ROI No. | mouse 1 | mouse 2 | mouse 3 | mouse 4 | mouse 5 | mouse 6 | mouse 7 |
| --- | --- | --- | --- | --- | --- | --- | --- |
| 0 | 1.18685 | 1.28548 | 1.23138 | 1.18604 | 1.26953 | 1.01131 | 1.22275 |
| 0.35 | 1.16616 | 1.21793 | 1.17117 | 1.13932 | 1.21498 | 1.03033 | 1.15666 |
| 0.7 | 1.15089 | 1.19404 | 1.14833 | 1.09810 | 1.18418 | 1.07252 | 1.12530 |
| 1.05 | 1.12605 | 1.14882 | 1.11660 | 1.06208 | 1.12656 | 1.07669 | 1.10280 |
| 1.4 | 1.14414 | 1.12670 | 1.11705 | 1.06728 | 1.09562 | 1.08409 | 1.10979 |
| 1.75 | 1.17244 | 1.11481 | 1.11858 | 1.08613 | 1.08215 | 1.11428 | 1.11273 |
| 2.1 | 1.17295 | 1.10344 | 1.12284 | 1.08457 | 1.08820 | 1.11365 | 1.11720 |
| 2.45 | 1.18883 | 1.10900 | 1.12744 | 1.09632 | 1.09757 | 1.12246 | 1.12455 |
| 2.8 | 1.17444 | 1.11840 | 1.10951 | 1.09782 | 1.09771 | 1.14073 | 1.12279 |
| 3.15 | 1.17847 | 1.11856 | 1.10372 | 1.12510 | 1.08965 | 1.16309 | 1.13050 |
| 3.5 | 1.17530 | 1.10651 | 1.09980 | 1.12425 | 1.08842 | 1.17331 | 1.13302 |
| 3.85 | 1.18018 | 1.09287 | 1.10676 | 1.11850 | 1.08420 | 1.18354 | 1.13737 |
| 4.2 | 1.17315 | 1.07671 | 1.10760 | 1.11625 | 1.07527 | 1.17886 | 1.11979 |
| 4.55 | 1.15326 | 1.06273 | 1.09530 | 1.11342 | 1.06805 | 1.16609 | 1.10305 |
| 4.9 | 1.13672 | 1.04832 | 1.07697 | 1.10624 | 1.05748 | 1.15820 | 1.09466 |
| 5.25 | 1.09195 | 1.02907 | 1.05390 | 1.08670 | 1.04202 | 1.14174 | 1.07350 |
| 5.6 | 1.05362 | 1.00279 | 1.02740 | 1.05680 | 1.02375 | 1.10552 | 1.03005 |
| 5.95 | 1.00085 | 0.97537 | 1.00643 | 1.03174 | 1.00561 | 1.08573 | 0.98744 |
| 6.3 | 0.96084 | 0.94335 | 0.97479 | 1.00939 | 0.97601 | 1.03334 | 0.95789 |
| 6.65 | 0.89549 | 0.91111 | 0.92912 | 0.96522 | 0.94356 | 0.99331 | 0.90539 |
| 7 | 0.85997 | 0.88475 | 0.90215 | 0.92109 | 0.91838 | 0.94750 | 0.88068 |
| 7.35 | 0.80718 | 0.85392 | 0.86749 | 0.87670 | 0.86525 | 0.89624 | 0.84066 |
| 7.7 | 0.75239 | 0.82478 | 0.83108 | 0.84695 | 0.82996 | 0.81995 | 0.81242 |
| 8.05 | 0.71030 | 0.79446 | 0.79372 | 0.81573 | 0.80234 | 0.78273 | 0.78289 |
| 8.4 | 0.67947 | 0.77074 | 0.76392 | 0.77712 | 0.78238 | 0.71335 | 0.76392 |
| 8.75 | 0.62541 | 0.73844 | 0.71634 | 0.72075 | 0.73491 | 0.64520 | 0.73106 |
| 9.1 | 0.56867 | 0.70402 | 0.67174 | 0.65586 | 0.70350 | 0.54810 | 0.69158 |
| 9.45 | 0.51403 | 0.64287 | 0.60885 | 0.61452 | 0.65276 | 0.49815 | 0.62954 |

Table S6. Fold induction of bioluminescence signals by KA in Fig.5c

| Sample | 0 h | 3 h | 6 h | 12 h | 24 h |
| --- | --- | --- | --- | --- | --- |
| saline 1 | 0.85844 | 1.11618 | 1.43099 | 1.05748 | 0.89493 |
| saline 2 | 1.20948 | 1.33491 | 1.55134 | 1.32882 | 1.12424 |
| saline 3 | 0.93209 | 1.26749 | 0.84165 | 1.25447 | 0.88965 |
| KA 1 | 0.91717 | 2.99011 | 1.86130 | 2.94130 | 1.94708 |
| KA 2 | 0.93030 | 3.62582 | 2.47529 | 3.84572 | 1.43104 |
| KA 3 | 1.15253 | 4.14639 | 2.59763 | 3.45256 | 1.32037 |

New animal mutant:

The *Arc-Luc* Tg HL mice will be available to the research community upon acceptance of this manuscript.
